# Anticancer properties of phospholipase A_2_ from *Daboia siamensis* venom on human skin melanoma cells

**DOI:** 10.1186/s40409-016-0061-z

**Published:** 2016-02-16

**Authors:** Suchitra Khunsap, Orawan Khow, Supranee Buranapraditkun, Sunutcha Suntrarachun, Songchan Puthong, Supatsorn Boonchang

**Affiliations:** Research and Development, Queen Saovabha Memorial Institute, Thai Red Cross Society, Bangkok, 10330 Thailand; Department of Medicine, Faculty of Medicine, Cellular Immunology Laboratory Allergy and Clinical Immunology Unit, Chulalongkorn University, Bangkok, 10330 Thailand; Institute of Biotechnology and Genetic Engineering, Chulalongkorn University Institute, Building 3, Phayathai Road, Patumwan, Bangkok, 10330 Thailand

**Keywords:** Phospholipase A_2_, *Daboia siamensis* venom, Skin melanoma, Anticancer

## Abstract

**Background:**

Phospholipase A_2_ (PLA_2_) is a major component of the *Daboia siamensis* venom, which is able to hydrolyse the membrane of various cells. For this reason, the activity of PLA_2_ was investigated regarding its pharmaceutical properties. This study was conducted to explore the pharmacological properties of a PLA_2_ from *Daboia siamensis* (dssPLA_2_) venom on human skin melanoma cell line (SK-MEL-28).

**Methods:**

dssPLA_2_ was isolated by ion exchange and gel filtration columns. Various concentrations of dssPLA_2_ were investigated for cytotoxic activity and inhibition of migration on SK-MEL-28 cells. Cell death analysis, mRNA expression levels of Notch I-III and BRAF V600E genes were also determined.

**Results:**

dssPLA_2_ exhibited cytotoxicity on SK-MEL-28 for 24 and 72 h as compared with untreated cells. However, it had no toxic effects on CCD-1064sk cells under the same conditions. dssPLA_2_ (0.25 and 0.5 μg/mL) induced 17.16 and 30.60 % of apoptosis, while activated 6.53 and 7.05 % of necrotic cells. dssPLA_2_ at 0.25, 0.5, 1 and 2 μg/mL could inhibit migration on SK-MEL-28 cells for 24 h by 31.06, 41.66, 50 and 68.75 %, respectively. The action of dssPLA_2_ significantly reduced the levels of Notch I and BRAF V600E genes expression on SK-MEL-28 cells compared with untreated cells at 72 h.

**Conclusions:**

This study indicates that dssPLA_2_ had potential effects of apoptosis, necrosis, cytotoxicity and inhibition of migration on SK-MEL-28 cells. dssPLA_2_ could possibly be a selective agent that targets cancer cells without affecting normal cells.

**Electronic supplementary material:**

The online version of this article (doi:10.1186/s40409-016-0061-z) contains supplementary material, which is available to authorized users.

## Background

Skin cancer, especially melanoma, is a medical problem with increasing incidence that is generally asymptomatic [1]. The risk factors for melanoma depends on several elements, including sun exposure, number of moles on the skin, skin type and family history [2]. Most malignant melanomas have mutation of the BRAF gene leading to constitutive activation of downstream signaling in the mitogen-activated protein (MAP) kinase pathway. The activating mutation consists of the substitution of glutamic acid for valine at amino acid 600 (V600E). Detection of BRAF V600E mutation gene bears relationship to survival and proliferation of cancer cells [3].

The surgical treatment in early stages of the disease is effective whereas treatment at late stages present low survival rates. Chemotherapy is a therapeutic option. However, its efficacy is limited due to chemoresistance and toxicity. Recently, new agents obtained from natural substances have been studied for their specificity against cancer cells and mild effects towards normal cells. For example, effects of rottlerin, involving the dual inhibition of ERK and NF-*κ*B and downregulation of cyclin D1, led to antiproliferation on SK-MEL-28 cells [4]. Gossypol, a cottonseed extract, has shown effective therapeutic action against BRAF V600E melanoma with resistance to BRAF inhibitors [5]. Three xanthones induced an inhibitory effect on SK-MEL-28 cell by modulating the BRAF V600E mutation and the other molecular targets in the apoptotic pathways [6]. Many small molecules have been studied in experimental and clinical trials. For example, vemurafenib, dabrafenib and trametinib, and serine/threonine kinase inhibitors have been tested in experiments and clinical trials as inhibitors for BRAF V600E mutation gene and MEK1/2 of MAPK pathway, respectively [7]. However, melanoma cell lines treated with these BRAF inhibitors have shown to rebound activation of phosphorylation of ERK (pERK) and escape from BRAF inhibition [8]. The progressed tumor returned within 6 to 8 months after therapy of these agents [9].

The disorder of Notch signaling pathways was associated with tumor genesis of tissues from various origins and also skin melanoma [10, 11]. Notch and their ligands are abundantly expressed in the epidermis, either as an oncogene or as a tumor suppressor on melanoma and non-melanoma [12]. The importance of Notch signaling pathway in determining the outcome of treatment has been reported [13]. Both BRAF V600E mutation and Notch signaling pathway should be studied as bioactive agents against melanoma.

PLA_2_ is one of the major components of snake venom for the digestion of prey [14]. Multifunctional effects of this enzyme have been documented [15, 16]. It directly catalyzes the hydrolysis of cellular phospholipids that generate lysophospholipids and free fatty acids that, in turn, cause membrane damage [17, 18]. Lysophospholipids are important in cell signaling, phospholipid remodeling and membrane perturbation [19]. Some evidence indicated that PLA_2_ hydrolyzed cellular membrane of various cancer cells [20]. In a study of membrane-interacted mode and catalytic activity, a PLA_2_ of *Naja naja atra* was able to catalyze outer leaflet and inner leaflet of plasma membrane-mimicking vesicles [21]. The venom from *Macrovipera lebetina transmediterranea* has shown to have antitumor effects mediated by α5β1 and αv-containing integrins [22]. Therefore, PLA_2_ properties indicated high activity against skin melanoma cancers.

Our previous study demonstrated the action of a PLA_2_ from *Daboia siamensis* (drsPLA_2_) against cancer cells [23]. In order to better understand the pharmacological action of dssPLA_2_ from *Daboia siamensis* against skin melanoma cells, the current work is focused on the process of migration inhibition, cytotoxicity and gene expression. Apoptosis analysis was also determined on skin melanoma cells. An investigation of molecular targets was performed to identify BRAF V600E mutation gene and Notch signaling receptors; N1, N2 and N3 genes.

## Methods

### Cell cultures

Human skin melanoma SK-MEL-28 (ATCC® HTB-72™) cells and skin fibroblast CCD-1064Sk (ATCC® CRL-2076™) cells were purchased from the American Type Culture Collection (ATCC, USA). SK-MEL-28 and CCD-1064sk cells were cultured in MEM and Iscove’s media supplemented with 10 % fetal bovine serum (FBS), 1 mM glutamine, 100 U/mL streptomycin and 100 U/mL penicillin. Cells were incubated at 37 °C with 5 % CO_2_. The media, FBS, streptomycin, penicillin and MTT 3-(4,-5-dimethylthiazol-2-yl)-2,5-diphenyltetrazolium bromide (MTT) were purchased from Invitrogen, USA.

### Venom separation by FPLC and SDS-PAGE

**Fig. 1:**
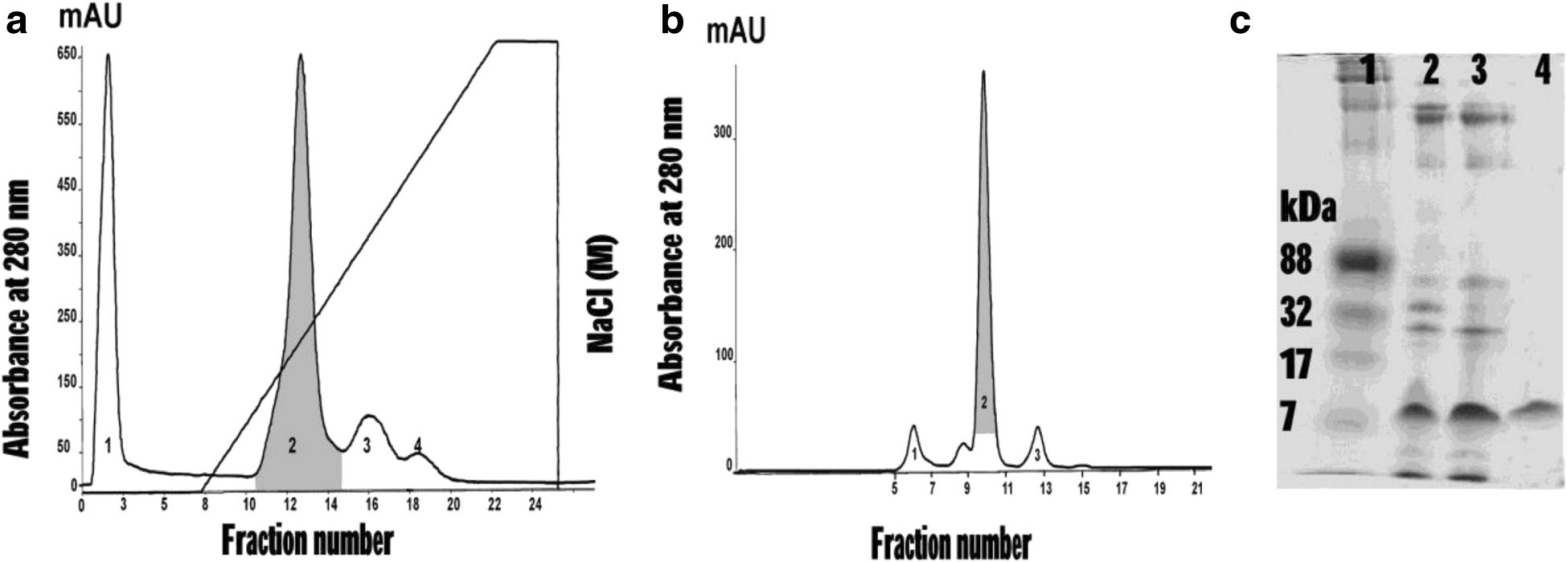
A dssPLA_2_ was isolated from *Daboia siamensis* venom. (**a**) Ion exchange chromatography of *Daboia siamensis* venom on HiTrap™ CM FF column. Protein was eluted with linear gradient 0–1 M NaCl. The peak with gray-shaded area was PLA_2_ activity. (**b**) Peak 2 from the first step was applied on Superdex™ 75 10/300 GL column. Elution was carried out with 10 mM PBS, pH 7.3. (**c**) SDS-PAGE analysis of venom and theirs fractions. Lane 1: protein markers, lane 2: crude venom, lane 3: fraction no.2 from ion exchange chromatography, and lane 4: fraction no. 2 from gel filtration chromatography

Crude venom from *Daboia siamensis* in lyophilized form was dissolved with 0.02 M phosphate buffer, pH 6.0. The venom solution (approximately 40 mg/mL) was applied to ion exchange chromatography on HiTrap^™^ CM FF column. The column was pre-equilibrated with 0.02 M phosphate buffer, pH 6.0. The proteins were eluted with 0–1 M NaCl linear gradient in 0.02 M phosphate buffer, pH 6.0, at a flow rate 0.5 mL/min. The proteins were collected and measured at absorbance 280 nm under an AKTA pure Fast Protein Liquid Chromatography system (FPLC, GE Healthcare; Sweden). Four peaks were observed and determined for phospholipase A_2_ activity. Peak 2, showed PLA_2_ enzyme activity (gray shaded area in Fig. 1a), was then further purified by size exclusion chromatography in a pre-equilibrated Superdex 75 10/100 GL column. Elution was carried out with 10 mM PBS, pH 7.3, at room temperature. The flow rate was adjusted to 0.5 mL/min., and 1 mL fraction was collected in each tube. The proteins were detected by absorbance at 280 nm under a Unicorn 6.3 Software. Four peaks were collected and tested for PLA_2_ activity. Peak 2 showed a PLA_2_ activity (gray shaded area in Fig. 1b) indicating a low molecular weight protein (below 17 kDa) on SDS-PAGE (Fig. 1c).

Each PLA_2_ fraction was analyzed on SDS containing polyacrylamide slab gels according to a modified method of Laemmli. The 12.5 % acrylamide in 3.0 M Tris–HCl, pH 8.8, was prepared in the presence of 0.1 % SDS. The venom samples (10 μg of protein) were mixed with a sample buffer and loaded onto a 12.5 % gel under non-reducing conditions. Electrophoresis was carried out at room temperature using 30 mA in the separating process. The precision plus protein standards were used as molecular weight markers. The electrophoresis was stopped when the marker dye reached the bottom of the gel. The electrophoresis gel was stained for 1 h with the Coomassie Brilliant Blue R-250 (0.2 % in methanol/water/acetic acid, 46.5: 46.5:7) and the excess stain was then removed by de-staining (methanol/acetic acid, 25: 12.5) with several changes of de-staining solution until the background was clear.

### Determination of molecular mass

The gel containing dssPLA_2_ was purified by a conventional method using a clean scalpel to cut off a strip of 15 % Tris-glycine SDS-PAGE. The sample was analyzed by mass spectrometry. Briefly, the excised gel was shredded and washed three times with 200 μL of 25 mM NH_4_HCO_3_/50 % acetonitrile (ACN). The gels were dehydrated with 200 μL of 100 % CAN, then rehydrated by 12.5 μg/mL of sequencing grade trypsin (Promega, USA) and incubated at 37 °C for 16 h. The supernatant was transferred to a new tube and added 100 μL of 50 % ACN/0.5 % formic acid. The mixture was finally dried by speedvac and suspended in 10 μL of 50 % ACN/0.1 % formic acid. The peptides were analyzed by MS/MS using microTOF-Q II^™^ ESI-Qq-TOF mass spectrometer (Bruker, Germany) equipped with an online nanoESI source. MASCOT is a software search engine that identifies proteins based on peptide sequence databases. Mass tolerance of parent and fragmented ions were 1.0 Da and 0.6 Da, respectively. MS/MS ions score ≥38 were considered significant hits.

### Phospholipase A_2_ activity

Phospholipase A_2_ activity was performed by Holzer and Mackessy method [24]. The sample (50 μL) was mixed with 3 mM 4-nitro-3-(octanoyloxy) benzoic acid 1:1 ratio (v/v) and incubated at 37 °C for 20 min. Triton X-100 (2.5 %) was added to the reaction mix and the absorbance was measured at 425 nm by ELISA reader. A standard curve of absorbance as a function of chromophore (3-hydroxy-4-nitrobenzoic acid) concentration showed that a change in absorbance of 0.10 AU at 425 nm was equivalent to 25.8 nmoles of chromophore release. The chromophore has an extinction coefficient of 5039 in this system. This sample was used as PLA_2_ fraction in the study of migration inhibition, apoptosis, cytotoxic activity, Notch signaling and BRAF V600E mutation genes expression on SK-MEL-28 cell line.

### Cytotoxic analysis

The MTT assay was chosen for determining the cytotoxic effect of venoms [25, 26]. Briefly, cells were seeded into 96-well plates (NUNC, Denmark) at concentration of 5 × 10^4^ cells/mL and incubated at 37 °C with 5 % CO_2_ for 24 h. The cells were treated with various concentrations of venom (0.25, 0.5, 1, 2 and 4 μg/mL) for 24 and 72 h. Cell viability was determined by adding 20 μL of MTT (2.5 mg/mL) for 3 h. An absolute DMSO (150 μL) was added to dissolve the formazan crystal. In the experiments, absorbance was measured at 540 nm and untreated cells were used for setting 100 % viability.

### Apoptosis analysis

SK-MEL-28 cells were seeded into 6-well plates and incubated at 37 °C with 5 % CO_2_ for 24 h. The dssPLA_2_ (0.25 and 0.5 μg/mL) were added then incubation continued for the next 24 houra. Apoptotic and necrotic cells were detected with AnnexinV apoptosis detection kit (USA), which is based on the observation after initial apoptosis. Cells translocate the membrane phosphatidylserine (PS) from the inner face of the plasma membrane to the cell surface. PS can be detected by staining with a fluorescent isothiocyanate (FITC) conjugate of AnnexinV while propidium iodide (PI) binds to the cellular DNA in necrotic cells. The staining process was performed following the manufacturer’s instructions. Cells were acquired and analyzed by FACSCalibur flow cytometry and CellQuest Pro software (Becton Dickinson, San Jose, USA).

### Inhibition of cell migration

Wound healing assay was performed for inhibition of cell migration [23]. SK-MEL-28 cells were seeded into 24-well plates (NUNC, Denmark) at 5 × 10^5^ cells/mL concentration and incubated at 37 °C with 5 % CO_2_ for 24 h. After 24 h, the cells were scratched and washed twice with medium without fetal bovine serum. The cells were treated with various concentrations of dssPLA_2_ and doxorubicin; an anticancer drug (2, 1, 0.5, 0.25 and 0 μg/mL) for 24 h. Inhibition of migration was determined by measuring the distance of the edge of scratching. The equation of migration inhibition was 100 – [((Z-Tn)/Z) × 100]. Z is the negative distance at time 0. Tn is the experiment distance at time 24 h.

### Real time-reverse transcription-PCR (qRT-PCR)

qRT-PCR was performed by following manufacturers’ recommendations (RBC Sciences). Total RNA was extracted from the SK-MEL-28 cells that were treated at 0.25 μg/mL of dssPLA_2_ for 24 and 72 h. Untreated of SK-MEL-28 cells were used as a negative control. One microgram of total RNA was employed to generate cDNA using random hexamer primers. One microliter of 1:10 cDNA dilution was applied to PCR reactions. Sequences of specific primers were Notch1 forward: 5’-CAGCCTGCACAACCAGACAGA-3’; reverse: 5’-TGAGTTGATGAGGTCCTCCAG-3’; Notch2 forward: 5’-AAAAATGGGGCCAACCGAGAC-3’; reverse: 5’-TCATCCAGAAGGCGCACA A-3’; Notch3 forward: 5’-AGATTCTCATCCGAAACCGCTCTA-3’; reverse: 5’-GGGGTCTCCTCCTTGC TATCCTG-3’; BRAF V600E forward: 5’-AGGTGATTTTGGTCTAGCTACAGA-3’; reverse: 5’-TAGTAACTCAGCAGCATCTCAGGG C-3’; β-actin forward: 5’-ACCAACTGGGACGACATG GACAA-3’; reverse: 5’-GTGGTGGTGAAGCTGTAGCC-3’.

qRT-PCR was performed based on SYBR Green (RBC Biosciences) to quantify the relative expression of BRAF V600E mutation gene and Notch signaling receptors: N1, N2, N3 genes. The mRNA level of the β-actin gene was detected in each cDNA sample to normalize the expression level of gene of interest (GOI). The ratio of GOI and β-actin was compared among samples. The fold change of GOI expression was acquired by setting the values from the untreated cells to one. Calculation of expression level of each gene that was normalized to the expression level of β-actin by Delta Delta Ct equation: ∆∆Ct = ∆Ct (Target gene treated – Reference gene treated) – ∆Ct (Target gene control – Reference gene control). The mRNA expression gene was calculated by 2 ^-(∆∆CT)^. CT was a threshold cycle.

### Statistical analysis

The statistical significance of the results was analyzed using *t*-test (Primer of Biostatistics version 3.02). Differences between the mean values of results were considered statistical significant at *p* < 0.05.

## Results

### Purification of phospholipase A_2_

In the process of purification, Peak 2 from the second step showed the highest PLA_2_ activity that increased 11.67 folds in the final step. The percent yield of dssPLA_2_ was 27.0 % of crude venom protein. The size of dssPLA_2_ was confirmed, 16.4 kDa, by using mass spectrometry. The summary of the purified method was displayed in Fig. 1. The purified protein was determined for biological activities.

### Cytotoxic analysis

**Fig. 2:**
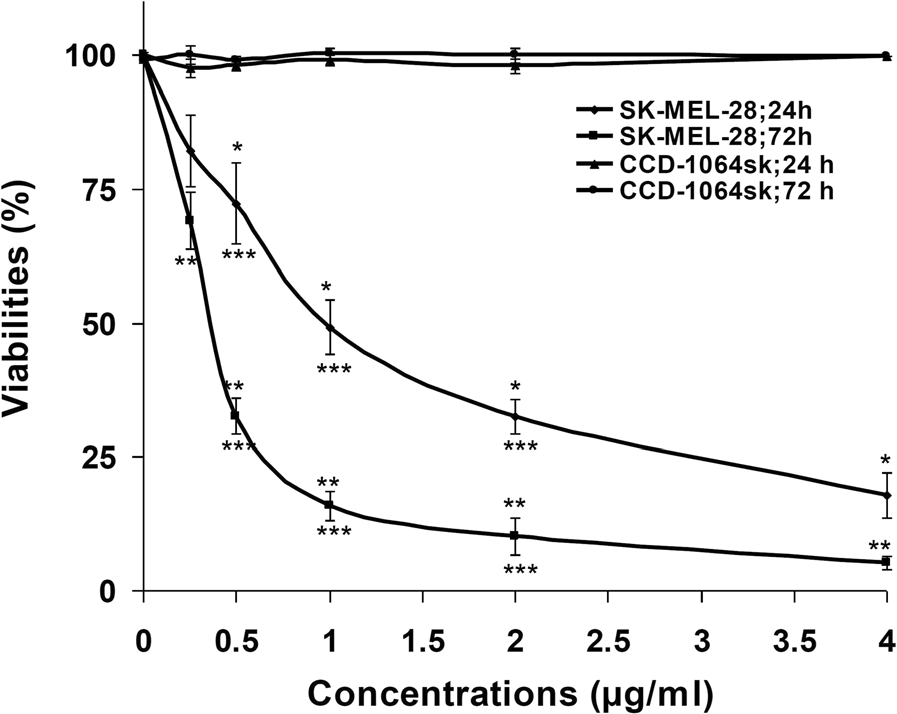
Viability of SK-MEL-28 and CCD-1064sk cells treated with dssPLA_2_ for 24 and 72 h. Data are expressed as means ± SE of triplicates (*n* = 4). Treatment that significantly differed from untreated control for 24 and 72 h at *p* < 0.05 are indicated as * and ** respectively. *** *p* < 0.05 indicates the significant differences between 24- and 72-h treatment at the same concentration

SK-MEL-28 and CCD-1064sk cells were treated with dssPLA_2_ at concentrations from 0 to 4 μg/mL for 24 and 72 h of incubation. dssPLA_2_ effects included dose- and time-dependent cytotoxicity on SK-MEL-28 cells. Viability of cells at 0.25, 0.5, 1, 2 and 4 μg/mL were, respectively, 82.26 % ± 6.73, 72.38 % ± 7.66, 49.19 % ± 5.09, 32.52 % ± 3.3 and 17.82 % ± 4.17 for 24 h whereas were 69.15 % ± 5.38, 32.65 % ± 3.42, 15.95 % ± 2.76, 10.23 % ± 3.35 and 5.3 % ± 1.21 for 72 h when compared with untreated cells. Viability of SK-MEL-28 cells at 72-h incubation was decreased approximately three fold at 1, 2 and 4 μg/mL and 1- and 2-fold at 0.25 and 0.5 μg/mL dssPLA_2_ concentration when compared with 24 h-incubation. However, dssPLA_2_ was not toxic to CCD-1064sk cells at the same conditions (Fig. 2).

### Apoptosis analysis

**Fig. 3:**
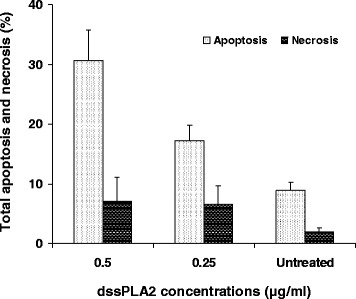
Total apoptosis and necrosis of SK-MEL-28 cells were induced by incubation with dssPLA_2_ for 24 h. Results are expressed as mean ± SE (*n* = 4)

dssPLA_2_ at 0.25 and 0.5 μg/mL induced, respectively, 17.16 and 30.60 % apoptosis in SK-MEL-28 cells. However, dssPLA_2_ also activated necrotic cells at 0.25 (6.53 %) and 0.5 μg/mL (7.05 %), while untreated SK-MEL-28 cells showed signs of necrosis in 1.92 % of the cells (Fig. 3). The results suggest that necrotic cells increased when incubation time and concentration of dssPLA_2_ augmented.

### Inhibition of cell migration

**Fig 4:**
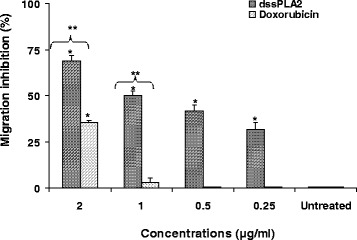
Inhibition of SK-MEL-28 cell migration by dssPLA_2_ from *Daboia siamensis* venom compared with doxorubicin (anticancer drug) for 24 h. Results are expressed as mean ± SE (*n* = 4). Significant differences between untreated and treated cells are presented as *p < 0.001. ** *p* < 0.001 indicates significant differences between dssPLA_2_ and doxorubicin at the same concentration

Wound healing assay was a simple tool to detect inhibition of SK-MEL-28 cells migration. The dssPLA_2_ inhibited migration on SK-MEL-28 cells by 31.06 % at 0.25 μg/mL, 41.66 % at 0.5 μg/mL, 50 % at 1 μg/mL and 68.75 % at 2 μg/mL after 24 h of incubation. However, more than 2 μg/mL of dssPLA_2_ did not increase inhibition of migration. Moreover, doxorubicin, an antineoplastic drug, inhibited less than 50 % of migration at concentrations up to 2 μg/mL (Fig. 4).

### Notch signaling and BRAF V600E mutation genes analysis

**Fig. 5:**
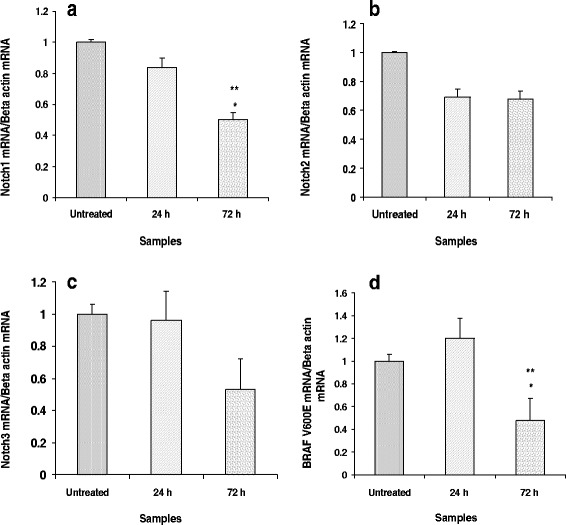
mRNA expression level of Notch1, Notch2, Notch3 and BRAF V600E mutation genes on SK-MEL-28 cells were induced by 0.25 μg/mL of dssPLA_2_ for 24 and 72 h. The values represent relative gene expression compared to untreated cells. Significant differences between untreated and treated cells are presented as *p < 0.001. ** *p* < 0.001 indicates difference between 24- and 72-h treatment at the same concentration

Effects of dssPLA_2_ to Notch signaling pathway and BRAF V600E mutation on SK-MEL-28 cells used real-time PCR as a tool for gene expression analysis. SK-MEL-28 cells were treated with 0.25 μg/mL dssPLA_2_ after 24 and 72 h of incubation. There was no significant change in mRNA expression level of genes on SK-MEL-28 cells between treated and untreated cells for 24 h. N1 and BRAF V600E mutation genes showed significant decrease of mRNA expression level on SK-MEL-28 cells between treated and untreated cells for 72 h (Fig. 5). The dssPLA_2_ did not significantly affect N2 and N3 genes.

## Discussion

Snake venom PLA_2_ are low molecular weight enzymes that have different isoforms depending on their geographical source and species of snakes [27]. In the present study, the action of dssPLA_2_ damaged SK-MEL-28 cells by causing cytotoxicity, apoptosis and inhibition of cell migration at 24 h and gene damage after 72 h of incubation. The dssPLA_2_ showed no toxicity towards normal skin cells (CCD-1064sk) by MTT assay. These results agree with a previous work that suggested that venom toxins specifically targets cancer cells without affecting normal ones [23]. It indicates a difference of cell membranes between cancer and normal cells, which reveals the variation of phospholipid domains in different cell types [14, 18, 28, 29].

Apoptosis is a programmed cell death that occurs as a defense mechanism when cells are irrecoverably damaged. Either lost of apoptosis function or the imbalance between cell division and cell death happen in case of cancer. Therefore, the induction of apoptosis pathway is a way to control the proliferation and inhibition of cancer progression. The effect of dssPLA_2_ (0.25 and 0.5 μg/mL) on apoptosis (17.6 % and 30.60 %) at 24 h of incubation suggests that dssPLA_2_ induced moderate apoptosis on SK-MEL-28 cells. Apoptosis pathway contains multiple cascades that may be investigated by various techniques. AnnexinV-FITC is a tool to detect phosphatidylserine (PS) on the cell surface in initiating apoptosis. Optimization of concentration and incubation period of dssPLA_2_ in order to provoke apoptosis on SK-MEL-28 cells could be improved in further studies. There were a couple of effects of dssPLA_2_ on SK-MEL-28 cells at 24 h of incubation. First, dssPLA_2_ induced apoptosis on SK-MEL-28 cells. In that moment, it had also provoked 31.60, 41.66, 50.0 and 68.75 % of migration inhibition at the following concentrations: 0.25, 0.5, 1 and 2 μg/mL. dssPLA_2_ (0.25 and 0.5 μg/mL) had induced death on SK-MEL-28 cells (23.19 and 37.65 %) at 24 h. The remaining surviving cells (76.81 and 62.35 %) resisted apoptosis and necrosis, and could migrate. Therefore, the cells migration might be inhibited due to other effects of dssPLA_2_. The minimum concentration of SK-MEL-28 cells (1 × 10^5^ cells/mL) could close a scratch within 24 h without inhibitors (Additional file 1). The results indicated that dssPLA_2_ had different actions in apoptosis and inhibition of migration on SK-MEL-28 cells at 24 h. However, both effects of dssPLA_2_ should be studied further.

One of the treatments for metastatic melanoma is based on BRAF V600E mutation gene. The BRAF V600E mutation gene has been reported to promote cancer cell survival and proliferation. This is found in most malignant melanomas [6, 30, 31]. Many selective small molecule inhibitors of BRAF V600E have been studied. Similarly to vemurafenib and dabrafenib, BRAF V600E mutation gene inhibitors have a limitation by frequent and rapid onset of resistance [4, 8, 9, 32].

In our study, qRT-PCR was used to determine the mRNA expression of BRAF V600E mutation on SK-MEL-28 cells treated by dssPLA_2_, compared with untreated control. In melanoma cancer, the mutation of BRAF V600 induced the hyperactive BRAF kinase activity and led to promote the uncontrolled cell proliferation [32]. The BRAF V600E mutation gene was a targeting to melanoma cancer treatment. Therefore, we examined the effect of dssPLA_2_ on BRAF V600E mutation gene to melanoma therapy. Moreover, Notch signaling genes, which are the cell cycle regulation genes, were also investigated in the same experiment. The Notch signaling pathway plays an important role in balance on the cell proliferation, differentiation and apoptosis pathways. Dysfunction in Notch pathway is connected with tumor mutagenesis in various tissues [12, 13, 30]. When those genes were blocked, the characteristic cell death would appear and dssPLA_2_ might be useful in treatment of this cancer.

The mRNA expression level of N1, N2, N3 and BRAF V600E genes between treated and untreated SK-MEL-28 cells were not significantly changed after 24 h incubation (Fig. 5). This result indicated that dssPLA_2_ had no effect of N1, N2, N3 and BRAF V600E mutation genes and were not related to induction of apoptosis and inhibition of cell migration. In contrast, the significantly decrease in the mRNA expression level of N1 and BRAF V600E mutation genes were observed on SK-MEL-28 cells after 72 h of incubation with dssPLA_2_ compared with untreated cells (Fig. 5a and d). This suggested that N1 and BRAF V600E genes may be connected with induction of cytotoxic process by dssPLA_2_ due to the mortality on SK-MEL-28 cells. A number of studies have examined the potential of Notch signaling genes by various agents. The results of these studies showed that down regulation of Notch signaling genes are similar to using dssPLA_2_. For example, down regulation of Notch1 provoked inhibition of cell growth and apoptosis in cancer cells [10, 11]. In contrast, the increased expression of Notch components was associated with a worse prognosis for cancer and a shorter survival rate [13]. Synthetic substances and natural products have been studied in order to find a melanoma inhibitor concerning this gene. For example, xanthones significant inhibited mRNA expression of BRAF V600E mutation gene at 48 h [6]. Numerous inhibitors – such as vemurafenib, dabrafebib and trametinib – are potential substances of interest since they target BRAF V600E and MAPK signaling. Unfortunately, the disease could re-emerge and escape from therapy. Therefore, the effect of dssPLA_2_ on melanoma should be evaluated in several aspects, especially resistance mechanisms focusing on BRAF V600E mutation gene and BRAF protein. dssPLA_2_ is a potential candidate against melanoma that would prevent the recurrence of the disease.

## Conclusions

We reported the effects of dssPLA_2_, a phospholipase A_2_ from *Daboia siamensis* venom, activity on SK-MEL-28 cells. This enzyme strongly inhibited migration and cytotoxicity and showed significant down regulation of N1 and BRAF V600E mutation genes on SK-MEL-28 cells that might lead to cell damage and death. Interestingly, it presented no toxicity towards CCD-1064sk, a normal skin cell.

## Ethics approval

This study was approved by the Queen Saovabha Memorial Institute Committee on the Ethics of Human Research (Reference number R2015-04).

## Additional file

Additional file 1:
**Amount of** **SK-MEL-28** **cells that could close a scratch within 24 h of incubation.** (PDF 5480 kb)

## Abbreviations

CCD-1064sk: Normal skin fibroblast cell line
DMSO: Dimethyl sulfoxide
dssPLA_2_: Phosphplipase A_2_ isolated from *Daboia siamensis* venom
FITC: Fluorescent isothiocyanate
FPLC: Fast protein liquid chromatography
MTT: MTT 3-(4,5-dimethylthiazol-2-yl)-2,5-diphenyltetrazolium bromide
N1, N2, N3: Notch 1, Notch 2, Notch 3
PI: Propidium iodide
PLA_2_: Phospholipase A_2_
PS: Phosphatidylserine
qRT-PCR: Quantitative reverse transcription polymerase chain reaction
SDS-PAGE: Sodium dodecyl sulfate polyacrylamide electrophoresis
SK-MEL-28: Human skin melanoma cells
