# Supplementary material for: Anticancer properties of phospholipase A2 from Daboia siamensis venom on human skin melanoma cells
Source: J Venom Anim Toxins Incl Trop Dis. 2016 Feb 16;22:7. doi: 10.1186/s40409-016-0061-z (PMC4754985; doi:10.1186/s40409-016-0061-z)
Supplement: Additional file 1: — Amount of SK-MEL-28 cells that could close a scratch within 24 h of incubation. (PDF 5480 kb) [file 40409_2016_61_MOESM1_ESM.pdf]

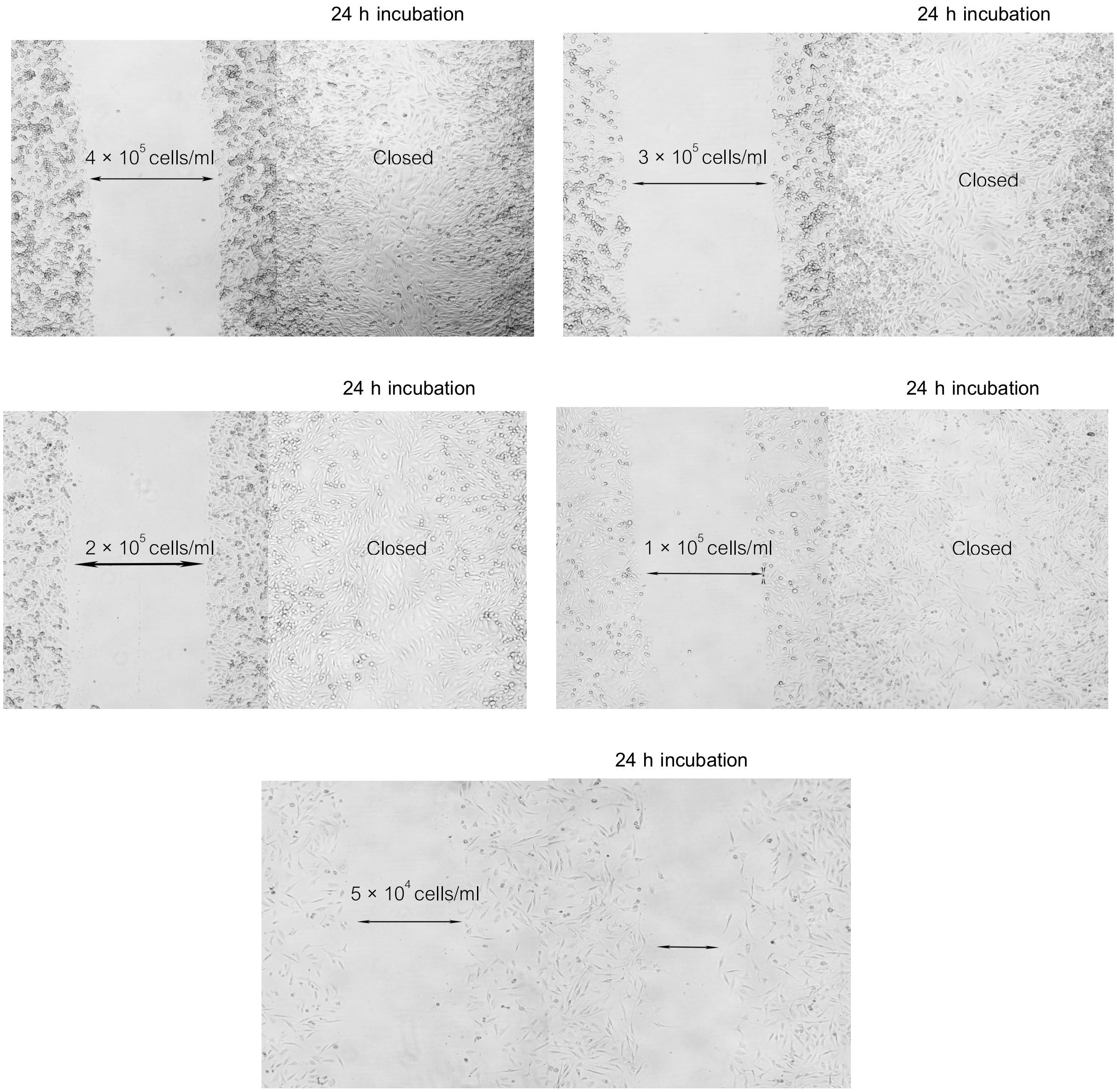

Additional file1. Amount of SK-MEL-28 cells closed within 24 h incubation after scratched.
